# Multifunctional Slippery Polydimethylsiloxane/Carbon Nanotube Composite Strain Sensor with Excellent Liquid Repellence and Anti-Icing/Deicing Performance

**DOI:** 10.3390/polym14030409

**Published:** 2022-01-20

**Authors:** Ke Liu, Chao Yang, Siyuan Zhang, Yao Wang, Rui Zou, Alamusi Lee, Qibo Deng, Ning Hu

**Affiliations:** 1 School of Mechanical Engineering, Hebei University of Technology, Tianjin 300401, China; lk516541003@163.com (K.L.); zsy550169073@outlook.com (S.Z.); bhwy2014@126.com (Y.W.); ruizou@hebut.edu.cn (R.Z.); alamusi@hebut.edu.cn (A.L.); qibodeng@hebut.edu.cn (Q.D.); 2 National Engineering Research Center for Technological Innovation Method and Tool, School of Mechanical Engineering, Hebei University of Technology, Tianjin 300401, China; 3 State Key Laboratory of Reliability and Intelligence Electrical Equipment, Hebei University of Technology, Tianjin 300130, China

**Keywords:** slippery strain sensor, liquid repellence, anti-icing/deicing, human motion monitoring

## Abstract

In this paper, a multifunctional slippery polydimethylsiloxane/carbon nanotube composite strain sensor (SPCCSS) is prepared using a facile template method. Benefitting from the slippery surface, the SPCCSS shows excellent liquid repellence properties, which can repel various liquids such as oil, cola, yogurt, hot water and some organic solvents. Meanwhile, the SPCCSS has a large strain sensing range (up to 100%), good sensitivity (GF = 3.3) and stable response with 500 cyclic stretches under 20% strain. Moreover, it is also demonstrated that the SPCCSS displays outstanding corrosion resistance (from pH = 1 to pH = 14) and anti-icing (8 min at −20 °C)/photothermal deicing (104 s with NIR power density of 1 W/cm^2^) properties, broadening its application in extreme acid, alkali and low-temperature conditions. Therefore, the multifunctional SPCCSS with the liquid repellence, anti-corrosion, and anti-icing/deicing properties has potential applications in wearable human motion monitoring tools under complex harsh environments.

## 1. Introduction

Flexible strain sensors possess excellent stretchability, flexibility and biocompatibility; therefore, it has wide potential application in human motion detection [1,2], healthy monitoring [3,4], and e-skin [5,6], etc. Generally, the flexible strain sensor is mainly made by conductive polymer composite, which consists of conductive fillers and polymer matrix. For instance, Zheng et al. prepared a flexible strain sensor by using a polydimethylsiloxane (PDMS), carbon nanotube (CNT) and carbon black (CB) mixed solution, and the strain sensor exhibited a wide sensing range (300%), high gauge factory (GF) of 13.1, and excellent stability over 2500 cyclic [7]. Wang et al. fabricated a flexible strain sensor by decorating the reduced graphene oxide (RGO) onto the thermoplastic polyurethane (TPU) fiber surface by ultrasonic treatment, which showed good sensitivity (GF = 79) and long-term durability (6000 cycles) [8]. Nevertheless, when the flexible strain sensor is exposed to severe environments including wet, corrosion, low temperature and so forth, the polymer matrix and conductive pathway are easily damaged, contributing to the decrease in sensing reliability of the strain sensor and even loss of the sensing property completely. To protect it from harsh environments, many important approaches have been put forward to construct a superhydrophobic flexible strain sensor, which has both low surface energy and large surface roughness with a water contact angle (CA) more than 150° and a sliding angle (SA) less than 10° [9,10,11,12,13]. For example, Preety et al. designed a superhydrophobic conductive composite by spraying the SiO_2_/PDMS solution onto the surface of the embedded strain sensor (PDMS/CNT/PDMS). The obtained strain sensor displayed excellent water resistance (CA = 161.5°) and good sensitivity (GF = 2.4) [14]. In addition, Sahoo et al. developed a superhydrophobic strain sensor by directly spraying a CNT solution onto a PDMS nanowrinkle substrate. The sensor possessed high CA of 165° and good response with over 5000 stretching-relaxing cycles [15]. These reports showed that the superhydrophobic strain sensor has the strain sensing characteristics under water interference, but the influence of corrosion liquid and temperature on the sensor performance was not considered. In view of this, Lin et al. reported a multifunctional superhydrophobic strain sensor based on sequential decoration of acid-modified carbon nanotubes (ACNTs), Ag nanoparticles (AgNPs) and PDMS onto the elastic TPU nanofiber surface. The sensor exhibited a high GF value of 1.04 × 10^5^, good corrosion resistance property (CA of 155° under acid, alikali and salt condition) and excellent deicing performance [16]. Furthermore, Wang et al. successfully prepared a superhydrophobic strain sensor by dipping the rubber band (RB) into Ag precursor solution to form an AgNPs shell modified with PDMS. The sensor showed extremely high sensitivity (GF = 3.6 × 10^8^), good anti-corrosion performance (CA of 154° with acid treatment for 8 h) and outstanding deicing performance with the applied voltage of 4 V [17]. However, the superhydrophobic structure of the above-mentioned strain sensors is fragile and can be easily contaminated by different types of liquid that have much lower surface tension [18,19], which severely restricts their actual applications. Inspired by the predation principles of a pitcher plant, the slippery lubricant infused surface that uses stable lubricant instead of the air layer between microstructures processes excellent performances of hydrophobicity, self-cleaning and anti-adhesion, meaning it has received extensive attention in the fields of anti-icing [20,21,22,23], anti-corrosion [24,25] and anti-fouling [26], etc. Therefore, endowing the strain sensors with a slippery surface is a promising strategy to protect the strain sensors from complex extreme conditions such as water phase, oil phase, corrosion liquid and low temperature.

Herein, we prepared a multifunctional slippery polydimethylsiloxane/carbon nanotube composite strain sensor (SPCCSS) by a silicon wafer template method. A uniformly dispersed PDMS/CNT mixture was poured into the silicon wafer template to form regular micro-pillar structured PDMS/CNT conductive polymer composite. By infusing the lubricant oil into the micro-pillars, the surface of the composite became considerably smooth, resulting in a slippery surface. The multifunctional SPCCSS exhibits excellent liquid repellency performance, which can repel various liquids such as oil, cola, yogurt, hot water and some organic solvents. Furthermore, it possesses a large strain sensing range (up to 100%), good sensitivity (GF = 3.3) and stable response with 500 cyclic stretches under 20% strain. Moreover, owing to the outstanding corrosion resistance and anti-icing/deicing properties, it can maintain the stability of the sensing performance under acid, alkali and low-temperature environments. To further test the practical application, the multifunctional SPCCSS was used to monitor the human body motions.

## 2. Experiments

### 2.1. Materials

PDMS (Sylgard 184) and silicone oil with a viscosity of 0.65–500 cSt was purchased from Dow Corning (Midland, MI, USA). Multi-walled carbon nanotube (CNT, diameter: 8–15 nm; length: 3–12 μm) were supplied by Suzhou Tanfeng Graphene Co., Ltd. (Suzhou, China). Perfluorinated silane was provided from Suzhou Yancai Micro Nano Technology Co., Ltd. (Suzhou, China). Xylene was obtained from Kelong Chemical Co., Ltd. (Chengdu, China). Krytox was bought from Dupont Holding Co., Ltd. (Shenzhen, China). Paraffin oil was purchased from Tianjin Chemical Three Plant Co., Ltd. (Tianjin, China). All chemicals were used as received without further purification.

### 2.2. Instruments and Characterizations

The morphology of the silicon wafer template and silicone oil-infused PDMS/CNT composite was characterized by optical microscope (Smartzoom, Zessis, Oberkochen, Germany). The microstructured PDMS/CNT composite was characterized by a scanning electron microscope (Merlin Compact, Opton, Beijing, China) with an acceleration voltage at 20 kV. The static contact angles and sliding angles were measured using a contact angle analyzer (JC2000D3M, Powereach, Shanghai, China) and the volume of liquid droplet that was used for measurements was 6 μL. An infrared thermal imager (T1040, FLIR, Portland, OR, USA) was used to record the photothermal conversion process under near-infrared laser (LSR808NL-3W-FC, Mingxi Laser, Ningbo, China) with different laser power densities. A digital camera (D7000, Nikon, Tokyo, Japan) was used to record the optical images of the melting process of frozen droplets. In order to study the strain sensing performance, the resistant (R, Ω) of the SPCCSS was measured by using a digital multimeter (DMM4050, Tektronix, Beaverton, OR, USA). The reproducibility and durability of the sensor were characterized by a universal electronic tensile machine (CMT6104, Xiangjie Instrument, Shanghai, China) through a cyclic tensile strain test.

## 3. Experiment Results and Discussion

### 3.1. Fabrication and Characterization of the Slippery PDMS/CNT Composite Strain Sensor

**Figure 1:**
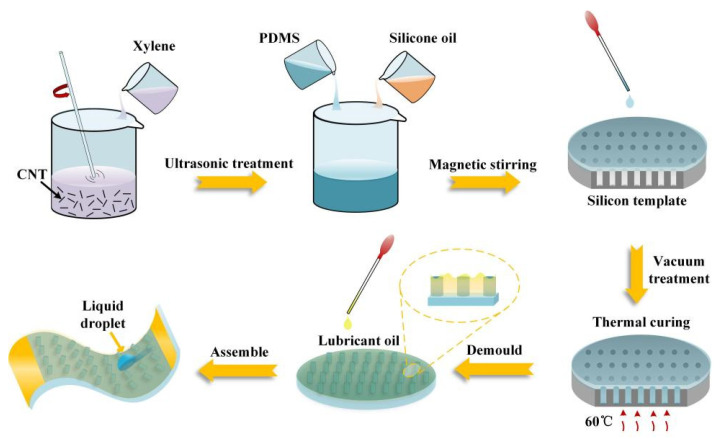
Schematic illustration of the fabrication procedure of the multifunctional SPCCSS.

The fabrication process of the SPCCSS is demonstrated in Figure 1. First, 1.5 g of CNT was ultrasonically dissolved in xylene solution for 20 min to form a dispersion solution uniformly, and then 15 g of PDMS and 8 g of silicone oil were added into the CNT solution. Meanwhile, the curing agent was dropped into the mixed solution to obtain a homogenous blend solution with magnetic stirring. Silicone oil was added to improve the stretchability of the PDMS/CNT composite, which was blended and distributed in the PDMS crosslinked network. Due to the swelling effect, silicone oil could separate PDMS chains and weaken the intermolecular interactions between PDMS chains, resulting in an improved mobility of PDMS chains [27]. Then, the PDMS/CNT solution was coated on the perfluorinated silane treated silicon wafer template, and vacuum treatment (−0.09 Mpa) was performed to ensure that the PDMS/CNT solution was fully immersed into the microporous structure. After being cured at 60 °C for 5 h and peeled from the template, the regular micro-pillar structured PDMS/CNT composite was obtained. Subsequently, the lubricant silicone oil was infused into the microstructured PDMS/CNT composite by vacuum impregnation (−0.09 Mpa) for 2 h. Finally, it was placed vertically for 8 h to remove excess silicone oil, and then two copper wires were tightly fixed on the two ends of the slippery PDMS/CNT composite with the help of copper tape and conductive silver paste.

**Figure 2:**
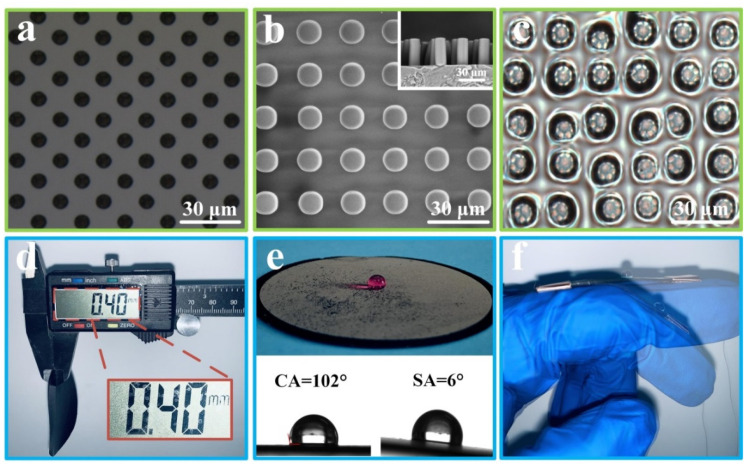
(**a**) Surface topography of the silicon wafer template. (**b**) SEM images of the surface structure of the PDMS/CNT composite after demoulding. (**c**) Photograph of the silicone oil-infused surface of the PDMS/CNT composite. (**d**) The thickness of the prepared slippery PDMS/CNT composite. (**e**) Optical image of the slippery PDMS/CNT composite and its water contact angle and sliding angle. (**f**) Optical image of the SPCCSS.

The surface morphology of the silicon wafer template prepared by the lithography method is shown in Figure 2a. The micropores with a diameter of 10 μm and depth of 30 μm are regularly arranged on the silicon wafer template, and the space between micropores is 10 μm. Figure 2b shows the SEM images of the PDMS/CNT composite after demoulding with different magnifications. It can be observed that the uniformly-sized micro-pillar structures are covered on the surface of the PDMS/CNT composite, and the size of the micro-pillars are highly consistent with the template, indicating that the microstructure of the template is successfully and completely duplicated to the PDMS/CNT composite. The surface morphology of the silicone oil-infused PDMS/CNT composite is illustrated in Figure 2c. It can be seen that large amounts of silicone oil are stored and filled in the gaps between micro-pillars, forming the slippery surface. More importantly, the silicone oil-infused slippery surface affords the PDMS/CNT composite excellent properties, such as liquid repellence, anti-corrosion and anti-icing, etc. In addition, as displayed in Figure 2d–f, the prepared SPCCSS possesses a pretty thin thickness (400 μm), which can be mounted onto the different parts of the body for monitoring human motion in real-time.

### 3.2. The Wetting Behavior and Liquid Repellence Property of the Slippery PDMS/CNT Composite Strain Sensor

**Figure 3:**
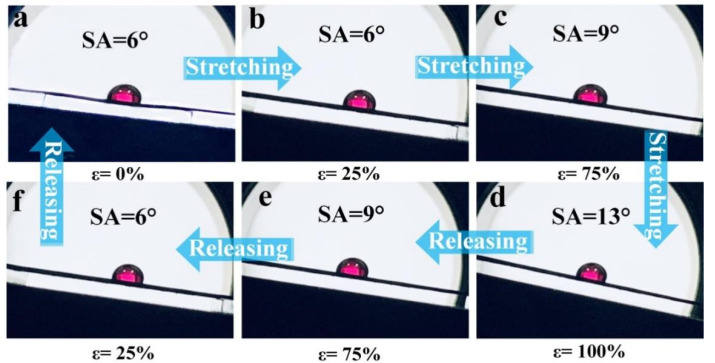
Images of the wetting behavior of the SPCCSS under different tensile states. The images of the SPCCSS at (**a**) ε = 0%, (**b**) ε = 25%, (**c**) ε = 75%, (**d**) ε = 100% during stretching and (**e**) ε = 75%, (**f**) ε = 25% during releasing.

The influence of the mechanical tensile strain on the wetting behavior of the slippery surface is demonstrated in Figure 3a–f. As the mechanical tensile strain increased from ε = 0% to ε = 100%, the SA of the slippery surface slowly increased from 6° to 13°. Notably, it could maintain excellent liquid repellency properties even when the strain reached 100%, and the water droplet slid easily at the SA of 13° (see Video S1 in the Supplementary Materials). Furthermore, the wetting behavior could be recovered to its initial state when the strain released back to 0%. The above phenomena can be demonstrated by the evolution of the surface morphology during stretching and releasing. When the composite is stretched, the space between the micro-pillars increases slightly in the stretching direction, which cancels out the decrease in micro-pillar depth. Therefore, the synergistic effect results in almost no significant increase in the SA of the slippery surface under large strain.

The effect of lubricant oil viscosity and types on the wetting behavior of the SPCCSS was further investigated (see Figure S1 and Video S2 in the Supplementary Materials), and it reveals that the wetting behavior of the slippery surface depends on the viscosity and types of lubricant oil, which results in the excellent liquid repellence property of the slippery surface. As demonstrated in Figure S1, both low and high viscosity liquids slide off the slippery surface easily, such as cola, tea, milk, and yogurt. Moreover, the slippery surface could also effectively repel the hot water and some organic solvents, such as ethylene glycol, formamide and butylene glycol. More details are shown in Video S3. Therefore, the SPCCSS can meet the requirements of different extreme conditions by infusing with different characteristics of lubricant oil and is expected to improve the application range of the SPCCSS.

### 3.3. The Anti-Icing/Deicing Property under Low Temperature

**Figure 4:**
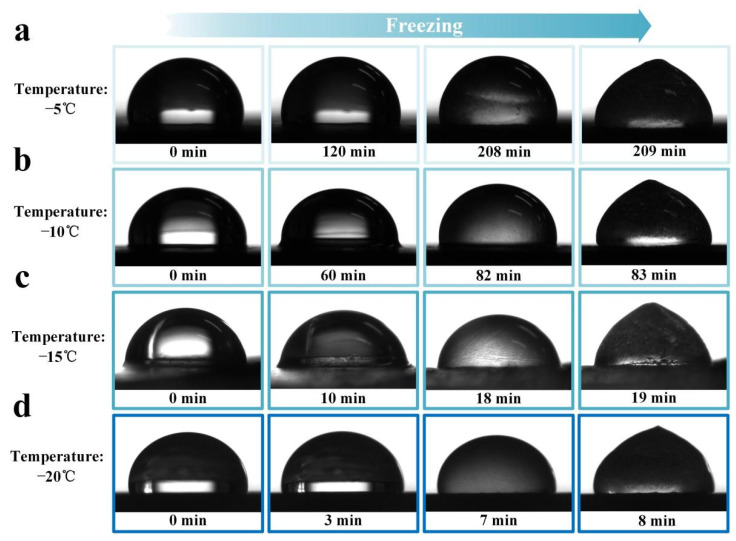
The images of the icing delay behavior of the SPCCSS at different temperatures: (**a**) −5 °C, (**b**) −10 °C, (**c**) −15 °C and (**d**) −20 °C.

The ice accretion on the surface has an extreme influence on the performance of the flexible strain sensor, especially on the sensing stability, which also brings inconvenience to the use of flexible strain sensors in low-temperature environments. The lubricant oil layer of the slippery surface can effectively isolate the droplet from direct contact with the substrate material, reducing the heat exchange between them, thus the time of icing is prolonged [28]. The icing delay time of the SPCCSS was tested, as shown in Figure 4. For the test, the water droplet of 6 μL on the surface was cooled down to different temperatures (−5, −10, −15 and −20 °C). The results showed that the slippery surface could delay the icing time to 209 min and 83 min at −5 °C and −10 °C, respectively, while 19 and 8 min for −15 and −20 °C, which provided sufficient time for the droplet sliding off the surface with the help of external force before freezing.

**Figure 5:**
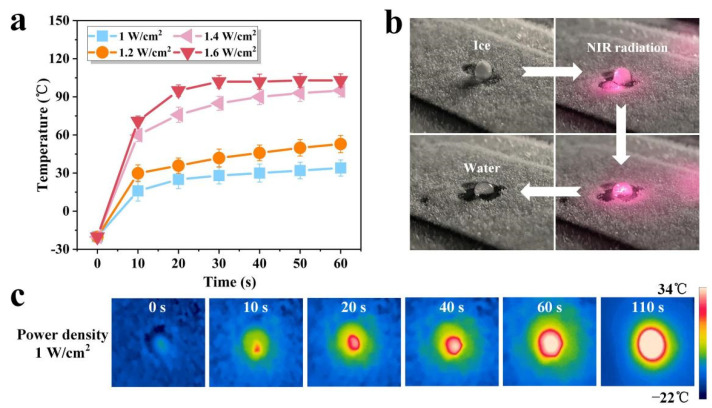
(**a**) Photothermal behavior of the SPCCSS under NIR with different power densities. (**b**) The ice melting process under the action of NIR with a power density of 1 W/cm^2^. (**c**) Infrared thermographic photographs of the SPCCSS under NIR with a power density of 1 W/cm^2^.

In addition to the excellent anti-icing property, the SPCCSS also exhibits good photothermal performance. The photothermal property of the SPCCSS under different laser power density of 808 nm near-infrared (NIR) laser at a low temperature of −20 °C was explored. Obviously, it can be seen from Figure 5a that the surface temperature of the SPCCSS increases with the increase in the power density of the NIR laser. At a low laser power density of 1 W/cm^2^, the maximum surface temperature could reach 32 °C, which was further enhanced to 103 °C at the laser power density of 1.6 W/cm^2^. Therefore, the SPCCSS retains the high photothermal conversion efficiency of CNT well, resulting in good photothermal properties and hence deicing performance. The photothermal deicing test was conducted on the surface of the SPCCSS, as shown in Figure 5b. The frozen droplet was completely melted into water at 104 s under NIR laser power density of 1 W/cm^2^, and the entire photothermal deicing process can also be clearly observed in Video S4. Under NIR irradiation, the interface between the frozen droplet and composite surface melted first due to the photothermal effect of CNT. With continuous NIR irradiation, the heat was transferred to the surrounding surface and then melted the whole frozen droplet. The photothermal conversion process of the slippery surface under NIR laser power density of 1 W/cm^2^ was recorded by an infrared camera, as illustrated in Figure 5c. The surface temperature increased from −20 to 32 °C within 60 s and further reached 34 °C at 110 s and gradually transferred to the surrounding surface. Hence, such an excellent photothermal deicing performance indicates very promising application prospects for SPCCSS under low temperatures.

### 3.4. Sensing Performance of the Slippery PDMS/CNT Composite Strain Sensor

**Figure 6:**
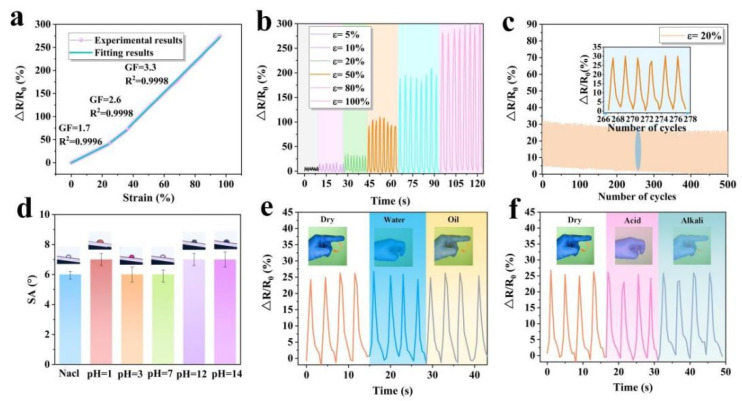
(**a**) Relative resistance change with tensile stretching and the linear fitting. (**b**) Strain sensing behavior of the SPCCSS at different strains. (**c**) Long-term sensing performance of the SPCCSS under a 20% strain for 500 cycles. (**d**) The SA of the SPCCSS under the salt and corrosion droplet with different pH values. (**e**) Relative resistance change with finger bending under water and oil droplet. (**f**) Relative resistance change with finger bending under acid and alkali droplet.

Except for the liquid repellence and anti-icing/deicing properties, the sensing performance of the SPCCSS was also studied. The mechanism of relative resistance change can be explained by the separation phenomenon of CNT networks, where CNT gradually separate under tensile strain, leading to the destruction of the conductive network and the increase in resistance. Furthermore, the relative resistant variation ΔR/R_0_ (ΔR = R − R_0_, where R is the resistance in the stretching state, R_0_ is the initial resistance) of the SPCCSS increased linearly as the tensile deformation increased (Figure 6a). It could be divided into three stages according to the variation of the resistance with the strain, and the correlation coefficient (R^2^) of each stage is 0.9996, 0.9998 and 0.9998, respectively, indicating that more than 99% of the experimental data is compatible with the fitting data. In the first stage, the ΔR/R_0_ reached 43% when the strain increased to about 25% and the gauge factory (GF = (ΔR/R_0_)/ε) was calculated to 1.7. In the second stage, the ΔR/R_0_ of the SPCCSS increased to 82% at a tensile strain of 40%, and the GF was up to 2.6. As the strain further increased to 100%, the ΔR/R_0_ increased by approximately 279% and the GF was measured to 3.3 in this stage. The ΔR/R_0_ was measured with a different periodic applied strain of 5%, 10%, 20%, 50%, 80%, and 100%, as shown in Figure 6b. It can be clearly seen that the peak value of ΔR/R_0_ increased synchronously with the increase in strain. When the periodic strain of 5% and 10% was applied, the corresponding periodic sensing signals increased to 7% and 15%, respectively, indicating that the SPCCSS possessed the characteristic of stable response even under low periodic strain. The ΔR/R_0_ increased by 35% as the strain reached 20%, and it rose to 114% when the strain further increased to 50%. Notably, under the large strain of 80% and 100%, the periodic electrical signals reached around 213% and 279%, respectively, and still could maintain a stable response. To examine the durability and stability of the SPCCSS, the 500 stretching-releasing cycles tests were carried out with the frequency of 0.5 Hz under the strain of 20%, as shown in Figure 6c. It can be seen that the sensing signal showed a stable change and was restored to the initial value under the cyclic strain of 20% except for the beginning stage of the cycle test, which is due to the hysteresis effect of PDMS. Consequently, the above results indicate that the SPCCSS possesses a great wide sensing range and sensing stability, which has great potential application in monitoring movement in different parts of the human body.

More importantly, the sensing performance of the SPCCSS under various droplets interference was investigated, as shown in Figure 6d–f. The SAs were kept around 6° under the salt and corrosion droplet of HCl and NaOH with different pH values from 1 to 14 (Figure 6d), which showed that the slippery surface could prevent the conductive network from being eroded by an external corrosion liquid. Furthermore, due to the outstanding liquid repellence and corrosion resistance properties, the sensing performance of the SPCCSS was not affected under various liquid droplets (water, oil, acid and alkali) dropping (Figure 6e,f). This is because the slippery surface isolates the external liquid droplets from the conductive network, and the silicone oil cannot be replaced by external liquid droplets due to its low surface energy. More details are shown in Video S5. Furthermore, in order to investigate the reproducibility of the sensor, four samples were prepared with the same fabrication process. Meanwhile, the sensing performance and wetting behavior of the sensors were further tested. The experimental results demonstrate that the samples remain highly consistent, indicating that it has a robust reproducibility, as demonstrated in Figure S2.

### 3.5. Application of the Slippery PDMS/CNT Composite Strain Sensor

**Figure 7:**
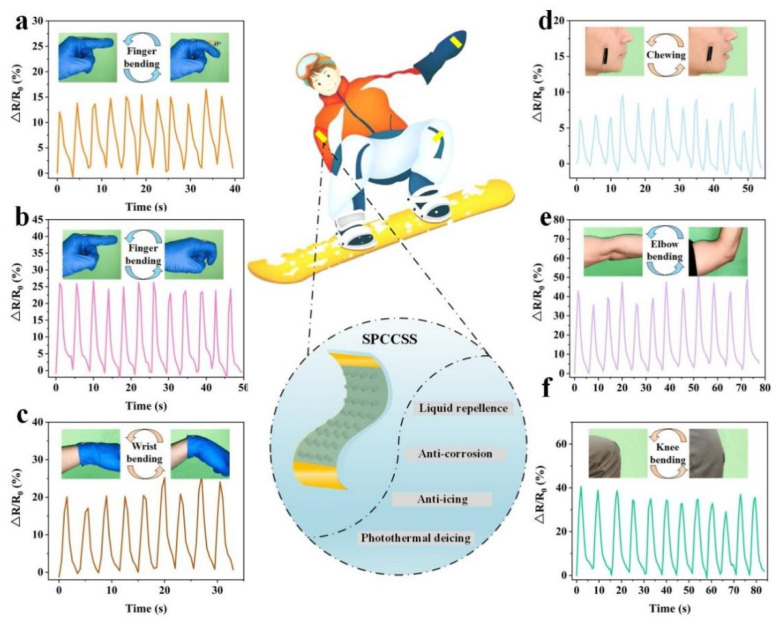
Monitoring of a variety of human motions using the SPCCSS: (**a**) Sensing signals of finger joint with a bending angle of 45°. (**b**) Sensing signals of finger joint with a bending angle of 90°. (**c**) Relative resistance response in detecting wrist bending. (**d**) Relative resistance response in detecting chewing movement. (**e**) Relative resistance variation of the sensor with elbow bending. (**f**) Relative resistance variation of the sensor with knee bending.

Owing to the good sensing stability, excellent liquid repellence properties, superior corrosion resistance and anti-icing/deicing performance of the SPCCSS, it can be used to detect full-range human body motions in winter sports such as skiing (Figure 7). As shown in Figure 7a,b, the SPCCSS was equipped on the knuckle of the index finger to monitor movements of varying degrees of flexion. The ΔR/R_0_ increased from 0% to 15% and around 25% when the finger bent from 0°, 45° and 90°, respectively, and then the ΔR/R_0_ returned to the initial value as the finger went back to 0°. Furthermore, the sensing signals of each cycle remained consistent, demonstrating the excellent repeatability. Similarly, other subtle body motions, including wrist bending and chewing movement, were also measured, and the corresponding variation of sensing signal was depicted in Figure 7c,d. In addition, the SPCCSS was also used to monitor the large motions, such as elbow bending and knee bending, as shown in Figure 7e,f. Obviously, the sensing signals also showed real-time changes as the variation of elbow and knee bending angles, and the ΔR/R_0_ increased to 50% and 60% with a bending angle of 90°. Therefore, the SPCCSS can be applied to monitor the movement of different parts of the human body in real-time, including large and subtle motions.

## 4. Conclusions

In summary, we have developed a multifunctional SPCCSS using a facile template method. By adjusting the viscosity and types of lubricant oil, the SPCCSS exhibits excellent liquid repellence performance even under large tensile strain (ε = 100%), which can repel various liquids such as oil, cola, yogurt hot water and some organic solvents. Furthermore, the SPCCSS possesses good sensitivity (GF = 3.3) and stable response with over 500 tension cycles under 20% strain. In addition, the SPCCSS has outstanding anti-icing (8 min at −20 °C) and photothermal deicing (104 s with NIR power density of 1 W/cm^2^) properties. Additionally, it can be used under various corrosion (from pH = 1 to pH = 14) and oil environments, which still maintain the stability of the sensing performance. Therefore, the multifunctional SPCCSS with the liquid repellence, anti-corrosion, anti-icing and deicing properties imply a promising application for human motion detection under complex harsh environments.

## Data Availability

Not applicable.

## Supplementary Materials

The following supporting information can be downloaded at: https://www.mdpi.com/article/10.3390/polym14030409/s1. Figure S1: (a) The variation in contact angles and sliding angles at different viscosities of the silicon oil. (b) The variation in contact angles and sliding angles at different types of lubricant oil. (c) Images of various liquids slide off the SPCCSS; Figure S2: (a) The sensing performance of different SPCCSS samples. (b) The wetting behavior of different SPCCSS samples.; Video S1: The water droplet slide down the slippery PDMS/CNT composite strain sensor at a sliding angle of 13° under the 100% strain.; Video S2: The wetting behavior of the slippery PDMS/CNT composite strain sensor.; Video S3: The various liquid droplets slide off the prepared slippery PDMS/CNT composite strain sensor.; Video S4: The photothermal deicing process of the slippery PDMS/CNT composite strain sensor.; Video S5: The sensing performance of the slippery PDMS/CNT composite strain sensor under the influence of the water droplet.
