# Supplementary material for: Multifunctional Slippery Polydimethylsiloxane/Carbon Nanotube Composite Strain Sensor with Excellent Liquid Repellence and Anti-Icing/Deicing Performance
Source: Polymers (Basel). 2022 Jan 20;14(3):409. doi: 10.3390/polym14030409 (PMC8838627; doi:10.3390/polym14030409)
Supplement: Supplementary file 1 [file polymers-14-00409-s001.zip › polymers-1540157-supplementary.pdf]

Supplementary Materials

Multifunctional Slippery Polydimethylsiloxane/Carbon Nanotube Composite Strain Sensor with Excellent Liquid Repellence and Anti-Icing/Deicing Performance

Ke Liu [a], Chao Yang [a*], Siyuan Zhang [a], Yao Wang [a], Rui Zou [a, c], Alamusi Lee [a, c], Qibo Deng [a], Ning Hu [b, c*]

[a] *School of Mechanical Engineering, Hebei University of Technology, Tianjin 300401, P.R. China*

[b] *State Key Laboratory of Reliability and Intelligence Electrical Equipment, Hebei University of Technology, Tianjin, 300130, P.R. China*

[c] *National Engineering Research Center for Technological Innovation Method and Tool, and School of Mechanical Engineering, Hebei University of Technology, Tianjin, 300401, P.R. China*

*Corresponding author

E-mail address: chao_yang@hebut.edu.cn (C. Yang), ninghu@hebut.edu.cn (N. Hu)

In order to investigate the surface wetting behavior of the SPCCSS, silicone oil with different viscosities (0.65, 10, 100, 250, 500 cSt) were used to infuse the micro-pillar structured surface, as shown in Figure S1a, it can be clearly seen that the lowest viscosity (0.65 cSt) silicone oil infused surface produces outstanding slippery property with the water SA value of 6° and water CA value of 102°. And, the SA of water droplet on the SPCCSS greatly increases with the increase of the silicone oil viscosity. For the silicone oil viscosity of 500 cSt, the SA increases to 16°. The high viscosity silicone oil exhibits solid-like character, and a large viscous drag is exerted on the sliding of the water, which leads to a growth in the SA.

In addition to the viscosity parameter, the type of lubricant oil used for preparation of the SPCCSS also played an important role in determining the wetting behavior of the slippery surface. As shown in Figure S1b, the micro-pillar structured surface infused with paraffin oil displays the SA of 19°, and SA value of perfluorinated polyether krytox 101 and krytox 103 are 16° and 25°, respectively. In addition, the different types of the lubricant oil has influence on the CA of the slippery surface (krytox 101 of 110°, paraffin oil of 107° and krytox 103 of 106°), which is attribute to the different surface energy of the lubricant oil.

As demonstrated in Figure S1c, the liquid repellency property of the SPCCSS was also investigated. As expected, both low and high viscosity liquids slide off the slippery surface easily, such as cola, tea, milk and yogurt. Moreover, the slippery surface could also effectively repel the hot water without heat-affected, and resist the

contamination of some organic solvents, such as ethylene glycol, formamide and butylene glycol. These droplets could remain hemispherical and slide rapidly on the surface of SPCCSS. This can be attributed to the low viscosity and low surface energy of the silicone oil layer, which prevents the external liquid contaminate the surface.

In order to investigate the reproducibility of the sensor, four samples (SPCCSS 1, 2, 3 and 4) were prepared with the same fabrication process. Meanwhile, the sensing performance and wetting behavior of the sensors were further tested and compared. As shown in Figure S2a, the $\Delta R/R_0$ of the four samples increased linearly as the tensile deformation increased and the tendency of the sensing curves remain highly consistent. In addition, the wetting behavior of the four samples is demonstrated in Figure S2b, it can be clearly seen that the SA of each sample were kept around 6°. Therefore, our approach showed a robust reproducibility.

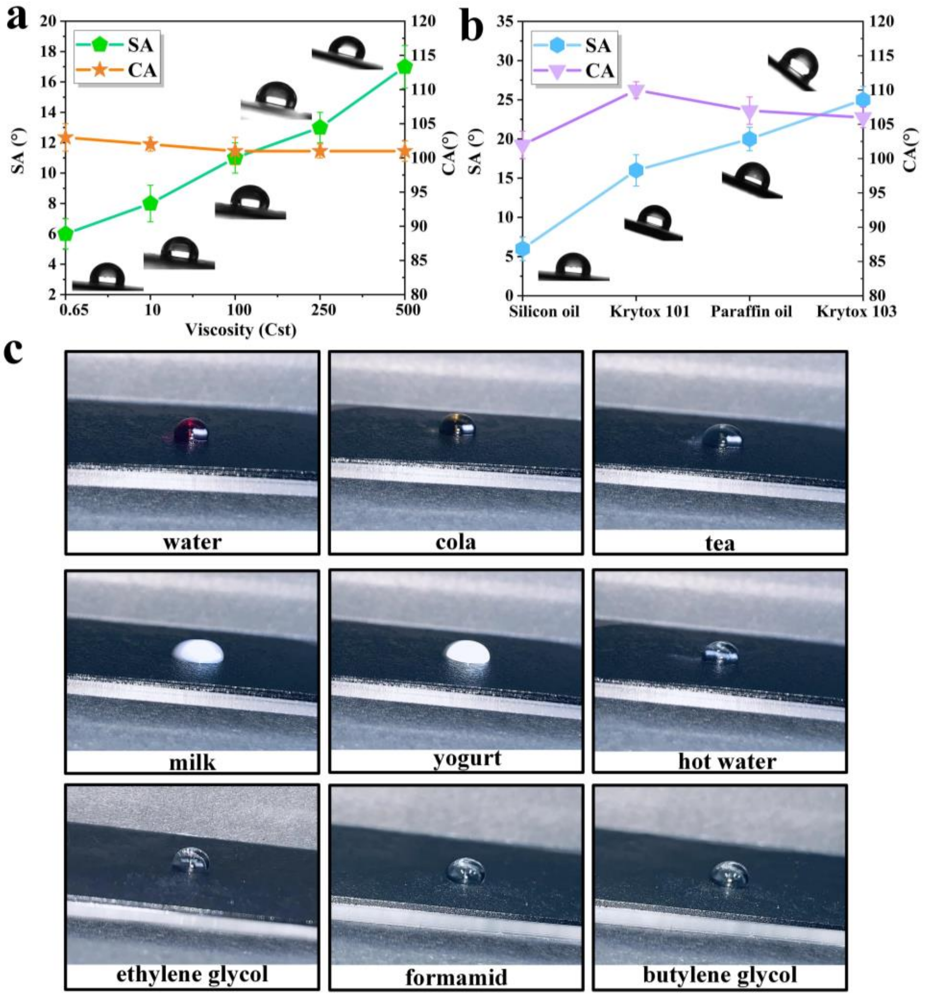

Figure S1. (a) The variation in contact angles and sliding angles at different viscosities of the silicone oil. (b) The variation in contact angles and sliding angles at different types of the lubricant oil. (c) Images of various liquids slide off the SPCCSS.

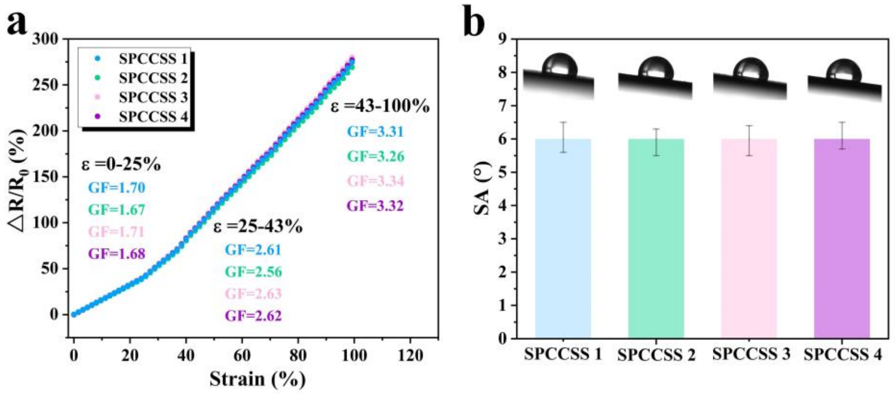

Figure S2. (a) The sensing performance of different SPCCSS samples. (b) The wetting behavior of different SPCCSS samples.

The water droplet slide down the slippery PDMS/CNT composite strain sensor at a sliding angle of 13° under the 100% strain. (Video S1)

The wetting behavior of the slippery PDMS/CNT composite strain sensor. (Video S2)

The various liquid droplets slide off the prepared slippery PDMS/CNT composite strain sensor. (Video S3)

The photothermal deicing process of the slippery PDMS/CNT composite strain sensor. (Video S4)

The sensing performance of the slippery PDMS/CNT composite strain sensor under the influence of the water droplet. (Video S5)
